# Association between Attention Deficit Hyperactivity Disorder and lower urinary tract symptoms in children and adolescents in a community setting

**DOI:** 10.1590/S1677-5538.IBJU.2020.0978

**Published:** 2021-05-20

**Authors:** Mônica Maria de Almeida Vasconcelos, José Murillo Bastos, Isaac Eduardo Arana, Isabela Benevenuto Teixeira, Eleonora Moreira Lima, Tânia Antunes Carvalho, José de Bessa, Flávia Cristina de Carvalho Mrad

**Affiliations:** 1 Universidade Federal de Minas Gerais Unidade de Nefrologia Departamento de Pediatria Belo HorizonteMG Brasil Departamento de Pediatria, Unidade de Nefrologia, Universidade Federal de Minas Gerais, Unidade de Nefrologia, Belo Horizonte, MG, Brasil; 2 Faculdade de Ciências Médicas e da Saúde de Juiz de Fora Departamento de Urologia Juiz de ForaMG Brasil Departamento de Urologia, Faculdade de Ciências Médicas e da Saúde de Juiz de Fora, Juiz de Fora, MG, Brasil; 3 Hospital e Maternidade Terezinha de Jesus Juiz de ForaMG Brasil Hospital e Maternidade Terezinha de Jesus, Juiz de Fora, MG, Brasil; 4 Universidade Estadual de Feira de Santana Departamento de Urologia SantanaBA Brasil Departamento de Urologia, Universidade Estadual de Feira de Santana, BA, Brasil

**Keywords:** Lower Urinary Tract Symptoms, Child, Attention Deficit Disorder with Hyperactivity

## Abstract

**Introduction:**

The present study aims to investigate the prevalence of lower tract urinary symptoms (LUTS) and symptoms of attention-deficit/hyperactivity disorder (ADHD) in children and adolescents and their association in a community setting using validated scoring instruments.

**Materials and Methods:**

A cross-sectional study was carried out from February 2015 to December 2019, during which the parents or guardians of 431 children and adolescents from 5 to 13 years of age, attending a general pediatric outpatient clinic were interviewed.

**Results:**

The prevalence of ADHD symptoms and LUTS were 19.9% and 17.9%, respectively. Of the 82 children and adolescents with ADHD, 28% (23) had LUTS (OR 2.31, 95% CI 1.28 to 3.75, p=0.008). Mean total DVSS score in children in the group of children presenting ADHD symptom was significantly higher than those without ADHD symptom (10.2±4.85 vs. 4.9±2.95, p=0.002). Urgency prevailed among LUTS as the most frequent symptom reported by patients with ADHD symptoms (p=0.004). Analyzing all subscales of the DVSS, the items “When your child wants to pee, can't he wait? “Your child holds the pee by crossing his legs, crouching or dancing?” were higher in those with ADHD symptoms (p=0.01 and 0.02, respectively). Functional constipation was present in 36.4% of children with LUTS and 20.7% without LUTS (OR 4.3 95% CI 1-5.3 p=0.001).

**Conclusion:**

Children and adolescents with ADHD symptoms are 2.3 times more likely to have LUTS. The combined type of ADHD was the most prevalent among them.

## INTRODUCTION

Attention-deficit/hyperactivity disorder (ADHD) is the most common neurodevelopmental condition and the second most frequent chronic disorder in children (1–3). ADHD is a clinical diagnosis defined as the occurrence of six or more symptoms both in the state of inattention and in the hyperactive/impulsive state or both, in children under 17 years of age (2, 4). Therefore, ADHD was categorized predominantly inattentive, predominantly hyperactive/impulsive, and combined types, representing 18.3%, 8.3% and 70% of ADHD children, respectively (4). ADHD is estimated to affect 5 to 10% of young people worldwide, being more common in boys than in girls (4–7). The Multimodal Treatment Study for ADHD of the Swanson, Nolan, and Pelham version IV (MTA-SNAP-IV) is a valuable instrument for assessing ADHD symptoms severity, besides being helpful for diagnosis purposes (8, 9).

Lower urinary tract symptoms (LUTS) are characterized by changes in the bladder emptying and/or storage phase and, if it there is coexistence with functional constipation it is named bladder bowel dysfunction (BBD) (10, 11). LUTS is present in about 21.8% of children and adolescents and girls are predominantly affected (12). The assessment of LUTS includes a careful clinical history and the use of validated questionnaires are helpful in identifying those presenting voiding symptoms. The Dysfunctional Voiding Symptom Score (DVSS) developed by Farhat et al. (13) and validated and adapted to our language and culture by Calado et al., 2010 (14) is considered one of the most commonly used instruments in evaluating LUTS and provides accurate and objective diagnosis of LUTS in children and adolescents.

ADHD and LUTS are not only common disorders in childhood, but also has a high co-existence and interaction with each other (15–18). The prevalence of ADHD in children and adolescents with LUTS is around 42.3% (19). Therefore, it is necessary to screen for ADHD symptoms in children and adolescents with LUTS (and vice versa) in order to improve treatment and, consequently, quality of life (11, 15, 16, 19).

We hypothesize that, in a general pediatrics clinics population, a significative association between these disorders can also be found. This study aims to investigate the prevalence of LUTS and ADHD symptoms in children and adolescents and the association between these two conditions in our population.

## MATERIALS AND METHODS

A prospective cross-sectional study was carried out from February 2015 to December 2019, during which 431 children and adolescents from 5 to 13 years of age attending a general pediatric outpatient clinic were evaluated. Children and adolescents with moderate to severe intellectual disability of any cause, urogenital malformation or diseases that may impair the function of the bladder or urethral sphincter, were not included in the study. A complete physical exam and standard assessment was performed on all subjects.

The study was approved by Institutional Review Committee (IRB), protocol number 2.625.013, and all parents or guardians of the patients signed an informed consent.

Gestational age at birth (premature less than or equal to 34 weeks, late preterm from 34 to 36 weeks, full-term) was investigated, based on the data recorded on the child's health card. The screening of ADHD symptoms and LUTS was performed through the application of MTA-SNAP-IV (20) and the DVSS (14) questionnaires adapted and validated for the Brazilian population. All interviews were conducted in a confidential environment by pediatricians trained for the application of the instruments, after evaluating the inclusion/exclusion criteria.

**Figure 1:**
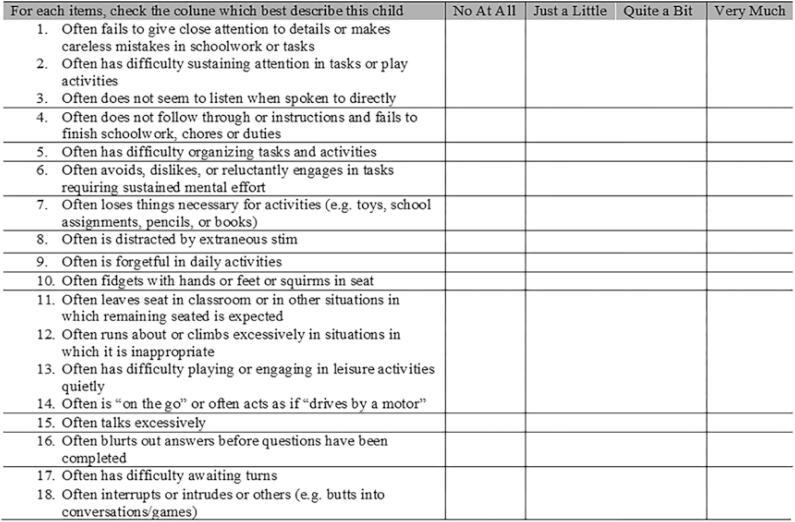
Multimodal Treatment Study for ADHD of the Swanson, Nolan, and Pelham version IV (MTA-SNAP-IV) adapted from Mattos et al. (20).

The MTA-SNAP-IV (19) includes two subscales with items related to inattention (items 1 to 9) and hyperactivity/impulsivity (10 to 18) and uses a 4-point Likert scale, ranging from 0 to 3 (0 Indicating nothing, 1 Just a little, 2 Quite a bit and 3 Very much) (Figure-1). The total score for each dimension is calculated by averaging the items (8, 20). If six or more items are marked as quite or very much in subscales 1 to 9, children or adolescents are considered to have more symptoms of inattention than expected. If six or more items are marked as quite or very much in subscales 10 to 18, children or adolescents are considered to experience more symptoms of hyperactivity/impulsivity than expected (20). All other individuals with scores below 6 on both subscales were classified as having no ADHD symptoms.

**Figure 2:**
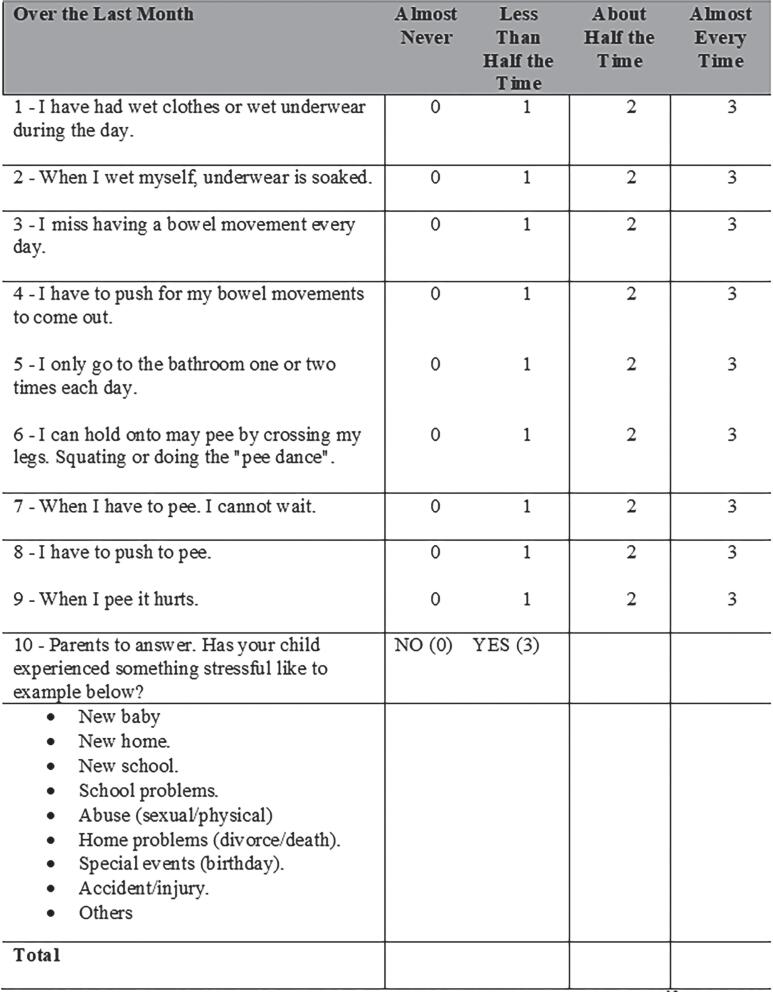
Dysfunctional Voiding Scoring System (DVSS) adapted from Fahart et al. (13) and Calado et al. (14).

Each subject, with the help of their parents or guardians, answered the ten questions of the DVSS questionnaire (14). The first nine questions assessed daytime incontinence, enuresis, constipation, urgency, voiding frequency, and dysuria. Scores were attributed on a scale of 0 to 3, with 0 indicating never or almost never, 1 less than half the time, 2 about half the time, and 3 almost every time. Question 10 assesses recent high stress events within the family and answers were dichotomic: yes, for a score of 3 and no for a score of 0. The cut-off value that indicates the presence of LUTS is >6 for girls and >9 for boys (13, 14) (Figure-2).

The presence of functional constipation was assessed according to the Rome IV criteria (21) (Appendix-1). The Bristol Stool Form Scale modified for children was used to evaluate stool consistency (22, 23) (Appendix-2).

Quantitative data was expressed as mean±standard deviation (SD) while qualitative variables were expressed as absolute values, percentages, or proportions. The Student t-test or the Mann-Whitney test was used to compare continuous variables, while the categorical variables were compared using the Fisher exact test. Odds ratio and 95% confidence intervals were used to describe the magnitude of association between LUTS and ADHD symptoms. All tests were 2-sided with p <0.05 considered statistically significant. Analysis was performed using commercially available statistical software (GraphPad Prism, version 8.03 for Windows, San Diego California USA).

All patients with confirmed LUTS and/or ADHD symptoms were referred for diagnosis, treatment and follow-up, in specialized outpatient clinics in these disorders.

## RESULTS

Four hundred twelve patients out of the 431 recruited were included in the study. Twelve parents refused to participate, three patients were diagnosed with severe intellectual disability, two had occult spinal dysraphism, and two had hypospadias with surgical complications. The mean age of participants was 7.26±1.84 years, being 53.4% males (220/412).

The overall prevalence of LUTS estimated by DVSS was 17.9% (74/412). Of those, fifteen (3.6%) of the 412 had the diagnosis of LUTS prior to the study.

ADHD symptoms were present in 19.9% (82/412) of children. Of those, a total of 24 patients (5.8% of 412) had neurodevelopmental disorders symptoms, specifically ADHD and six of them (25%) had a diagnosis and were receiving treatment with partial response. Of the patients presenting ADHD, 6.1% (5/82) had inattention type, 9.8% (8/82) hyperactivity/impulsivity type, and 84.1% (69/82) combined type of ADHD. When compared by gender, ADHD symptoms were present in 59.7% (49/82) of the boys (OR 1.4, 95% CI 1 to 2.9, p=0.003).

**Figure 3:**
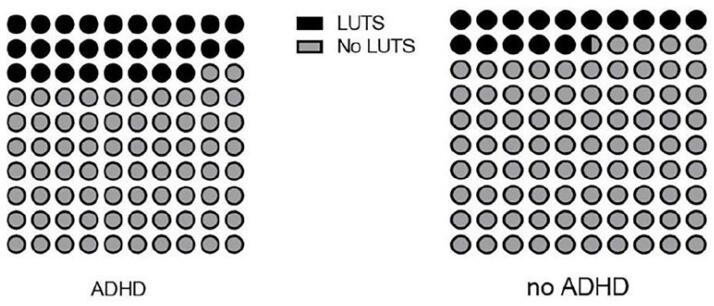
Prevalence of Attention-Deficit Hyperactivity Symptoms (ADHD) in Children and Adolescents with Lower Urinary Tract Symptoms (LUTS).

Of the 82 children and adolescents with ADHD symptoms, 28% (23/82) had LUTS (OR 2.31, 95% CI 1.28-3.75, p=0.008), being 56% (13/23) males. The combined type of ADHD was present in 91.3% (21/23) and hyperactivity/impulsivity type in 8.7% (2/23) of the subjects with LUTS (OR 7.5, 95% CI 1.5-4.78, p=0.001). None of those with the inattention type presented LUTS (Figure-3).

The average DVSS total score in children and adolescents with ADHD symptoms was significantly higher than in those without (10.2±4.85 and 4.9±2.95, respectively, p=0.002). Urgency was the most common LUTS, being more frequent in those with ADHD symptoms (65% versus 35%, p=0.004). When we analyzed the average score of all DVSS subscales, responses to the items “When your child wants to pee, can't he wait?” and “Does your child hold the pee by crossing his legs, crouching or dancing?” were higher in those with symptoms of ADHD (p=0.01 and 0.02, respectively) (Table-1).

**Table 1:** Description of Lower Urinary Tract Symptoms (LUTS) in Children and Adolescents with Attention-Deficit Hyperactivity (ADHD) Symptoms.

Characteristics	ADHD symptoms Mean ± SD		No ADHD symptoms Mean ± SD		p value
Age	7.4 ± 2.1		7.2 ± 1.8		0.8
Gender Male	59%		41%		0.03
DVSS Total	10.2 ±4.85		4.9± 2.95		0.02
Variables	Number Patients (%)	Mean Score ± SD	Number Patients (%)	Mean Score ± SD
	**Total 82**		**Total 330**		
1. Daytime incontinence	19 (23.7)	0.66 ± 0.8	58 (17.5)	0.62 ± 0.7	0.8
2. Soaked underwear	12 (14.6)	1.02 ± 0.6	36 (10.9)	0.9 ± 0.4	0.35
3. Frequency of evacuation	15 (18.2)	0.9 ± 0.54	69 (20.9)	0.7 ± 0.55	0.91
4. Push bowel movements	19 (23.1)	1.02 ± 0.63	60 (18.1)	0,85 ± 0.35	0.67
5. Low urinary frequency	11 (13.2)	0.86 ± 1.2	30 (9.09)	0.72 ± 0.99	0.3
6. Holding maneuvers	23 (28.0)	1.92 ± 1.2	38 (11.51)	0.82 ± 0.82	0.02
7. Urgency	26 (31.7)	2.11 ± 1.1	28 (8.48)	0.93 ± 0.74	0.01
8. Straining to void	12 (14.6)	0.62 ± 1.2	23 (6.96)	0.74 ± 1.1	0.3
9. Dysuria	6 (7.3)	0.33 ± 0.21	28 (8.48)	0.22 ± 0.44	0.79
10. Stressful events	18 (21.9)	1.33 ± 0.21	79 (23.9)	0.91 ± 0.17	0.07

Dysfunctional Voiding Symptom Score (DVSS); p<0.05

The overall prevalence of functional constipation (characterized according to the Rome IV criteria) was 31% (129/412), being 36.4% (27/74) with LUTS and 20.7% (70/338) in the ones without LUTS (OR 4.3 95% CI 1-5.3 p=0.001). Ninety-five percent of the individuals with functional constipation had stool types 1 and 2 of the Bristol Stool Form Scale modified for children. There was no statistical difference in the prevalence of functional constipation between those subjects with and without ADHD symptoms, both associated with LUTS (p=0.74).

Among the children who had symptoms of LUTS and ADHD, 78% (18/23) were full-term, and 22% (5/23) were premature (one with gestational age of less than 34 weeks) (p=0.6).

## DISCUSSION

The present study demonstrated that children with ADHD have 2.3 times more chance of presenting LUTS, and that the most common voiding symptom in this population is urgency. The overall prevalence of ADHD symptoms in this study was in accordance to that previously reported. A recent review showed variability in the worldwide prevalence of ADHD symptoms around 5 to 29% in community samples of children and adolescents. This variability in the prevalence of ADHD was attributed to methodological differences between the studies, specifically in the diagnostic criteria and sources of information between different countries (3). The observance of specific behaviors in various settings remains the most successful method for diagnosing ADHD (1, 3). Although there are differences in particular areas of the brain and a high estimate of heritability (about 76%), no test (neuroimaging or neurotransmitters) or genetic pattern is necessary or enough for the diagnosis of the disorder (1). Regarding ADHD subtypes, 84% of our sample were identified with the combined subtype, also in agreement with other studies (5, 9, 24).

In the present study, we have found a high prevalence of LUTS (28%) in children and adolescents with ADHD symptoms. Individuals diagnosed with ADHD symptoms by MTA-SNAP-IV questionnaire were more likely to have LUTS, been the combined type the most frequent type, while hyperactivity/impulsivity type present in less than 10% of the patients with LUTS. Contrasting with our findings, Crimmins et al. showed that children with hyperactivity/impulsivity type ADHD is approximately 4.5 times more likely to have LUTS (25).

A longitudinal study found that early childhood externalizing (as impulsivity and hyperactivity) and inattentive symptoms were associated with daytime urinary incontinence with increased odds of enuresis at 10 years and adolescents (26). Therefore, there is strong evidence in all age groups that ADHD is more common in patients with LUTS and vice versa. ADHD may be related to noradrenergic and dopaminergic pathways in the central nervous system, with decreased adrenergic activity affecting the lower urinary tract. Decrease in the _β_-adrenergic effect leads to contraction of detrusor, while an increase leads to relaxation of the detrusor (27). Regardless of the cause, it is a priority to address LUTS in patients of all age groups with neurodevelopmental conditions and vice-versa, using objective diagnostic tools including validated questionnaires (6, 28, 29).

Two instruments validated for the Brazilian population to assess LUTS (14) and ADHD (19) were used. The mean DVSS total score in the group with ADHD symptoms (10.2) was significantly higher than in the group without ADHD (4.9). Similar results were found by Yang et al. (19) and Burgu et al. (28). The urgency scores raised by the question 7 in DVSS (“When your child wants to pee, can't he wait?”) were significantly higher in the group with ADHD symptoms, similar to other studies (19, 28, 30). We also found a high prevalence of holding maneuvers in our series, elicited by question 6 in DVSS (“Your child holds the pee by crossing his legs, crouching or dancing?”), which, to our knowledge, hasn't been demonstrated yet.

The assessment of bowel habits is recommended as an approach for children and adolescents with LUTS to diagnose BBD (11, 12). In this study, functional constipation was detected in 36% of individuals with LUTS. These findings corroborate the results found by other authors (12, 30, 31). However, ADHD did not increase the chance of having constipation in those presenting LUTS, different from the finding of Crimmins et al., 2003 (25), who found that children and adolescents with ADHD symptoms are significantly more likely to have functional constipation and fecal incontinence (32).

Regarding gestational age, 78% of participants with LUTS and ADHD symptoms were full term. A recent study reported that prematurity is independently associated with the diagnosis of neurological development disorders. Also, it showed that 19.5% of premature infants have ADHD, with a prevalence inversely proportional to gestational age (33). No studies were found showing an association between prematurity and LUTS.

This study has some limitations. Due to its configuration and design, the patient teacher's report on the MTA-SNAP-IV data was not included, which could increase its screening power (9). Also, the instrument's application was not repeated, which would be important for consistency results. We seek to minimize this limitation with the appropriate training of professionals who applied the instruments during outpatient care. In addition, it was not possible to provide information on the causal links between the two conditions, due to the cross-sectional nature of the study. It is important to state that all study subjects were recruited from a general pediatric clinic. Therefore, urofluxometry with electromyography and voiding diary were not obtained.

On the other hand, some features of this study may increase the strength of our findings, such as sample size and the use of standardized questionnaires. Most studies examine risk factors for nocturnal enuresis, with very few studies examining daytime voiding symptoms.

## CONCLUSION

Children and adolescents, recruited in a general pediatric outpatient clinic, with ADHD symptoms are 2.3 times more likely to have LUTS. The combined type of ADHD was the most commonly associated with LUTS. Urgency and holding maneuvers were most prevalent symptoms in children and adolescents with ADHD symptoms. These findings support that all children with ADHD should be addressed for LUTS and vice versa.

## Figures and Tables

**Appendix 2:**
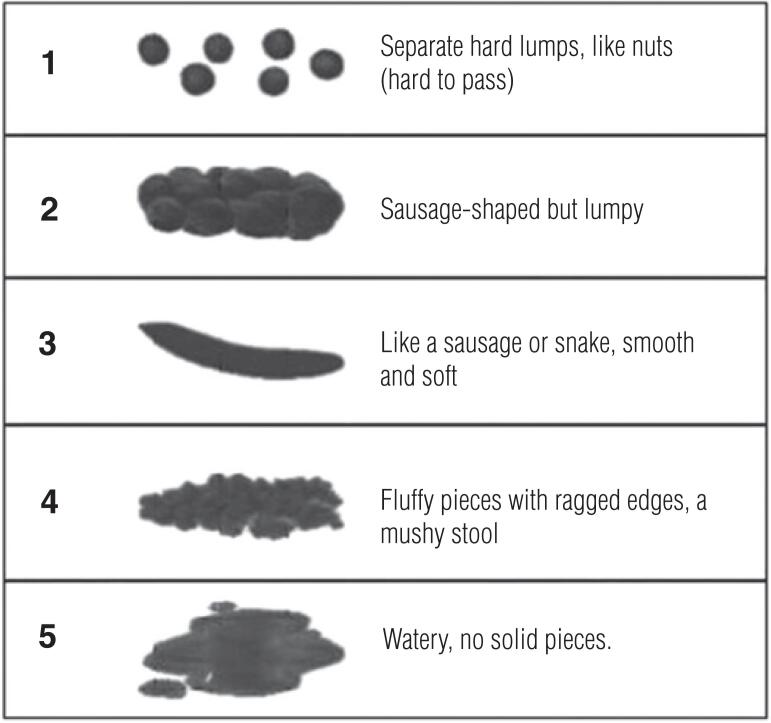
Modified Bristol Stool Form Scale for Children adapted from Lane et al. (22).

## APPENDIX

### Appendix

**Appendix 1:** Diagnostic Rome IV Criteria for Functional Constipation adapted from Benninga et al. (21).

Must include 1 month of at least 2 of the following in infants up to 4 years of age:
1. Two or fewer defecations per week
2. History of excessive stool retention
3. History of painful or hard bowel movements
4. History of large-diameter stools
5. Presence of a large fecal mass in the rectum
**In toilet-trained children, the following additional criteria may be used:**
6. At least 1 episode/ week of incontinence after the complete toilet training process
7. History of large-diameter stools that may obstruct
